# High Temperature Effects during High Energy Laser Strikes on Embedded Fiber Bragg Grating Sensors

**DOI:** 10.3390/s19061432

**Published:** 2019-03-23

**Authors:** Michael J. Ross, R. Brian Jenkins, Charles Nelson, Peter Joyce

**Affiliations:** 1 Department of Electrical and Computer Engineering, US Naval Academy, 105 Maryland Ave., Annapolis, MD 21402, USA; ross.michaelross.michael@gmail.com (M.J.R.); cnelson@usna.edu (C.N.); 2 Department of Mechanical Engineering, US Naval Academy, 590 Holloway Rd., Annapolis, MD 21402, USA; pjoyce@usna.edu

**Keywords:** Fiber Bragg Grating, FBG sensor, High Energy Laser (HEL), composite, spectrum, reflectivity, thermal decay

## Abstract

As the applications of fiber Bragg gratings (FBGs) continue to grow and become more advanced, it becomes necessary to understand their behavior when exposed to high temperatures in unique situations. In these experiments, uniform 1530-nm fiber Bragg gratings and Type K Cr-Al thermocouples were embedded in three-ply carbon fiber composites. A 100 W high energy laser (HEL) heated the composites to high temperatures over timespans less than one second, and FBG spectral data and thermocouple temperature data were collected during each HEL heating test. The data from three high energy laser tests that represent different levels of damage to the FBG are analyzed to explore the spectral response and thermal decay of embedded FBG sensors when exposed to high temperatures over short timespans. Results are compared to a previously proposed power-law model describing the decay of FBGs in bare fiber when held at constant temperatures over much longer timespans.

## 1. Introduction

Fiber Bragg grating (FBG) sensors have become an area of significant research interest due to their relatively small size, high sensitivity and quick response times as well as their immunity to electromagnetic interference [1]. FBG technology was first introduced in 1978 [2] and is now commonly used in communication applications and, more recently, in sensor applications [3]. FBG sensors are highly sensitive to small changes in axial strain, which is useful in many applications including embedded strain sensors in polymer and polymer-based composites [4,5,6], structural health monitoring in infrastructure [7,8] and airframe structures [9], and measuring the weights of moving automobiles [10]. FBG sensors are also responsive to changes in temperature, which makes them useful in applications such as process monitoring during pharmaceutical production [11] and health monitoring of steel structures [12], fires [13], oil pipelines [14], and laser strike detection [15,16]. This paper focuses on the temperature limits and thermal decay of embedded FBG sensors in such an application.

An accurate model of FBG thermal response and decay is necessary to assess the viability of implementing a FBG temperature sensor network, especially in harsh environments. The experiments outlined in this paper were conducted to analyze the behavior of embedded FBG sensors in high energy laser (HEL) sensing applications, where large, sudden, and non-uniform temperature transients are possible. There are several examples of high-temperature FBG experiments in recent literature, including studies of FBG decay at constant temperatures during annealing [17,18], FBG spectral shift as a function of temperature and laser intensity [19], and the performance of an array of embedded FBG sensors during an HEL strike [16]. Additionally, more recent work by Jenkins et al investigated embedded distributed optical fiber sensors for use in laser strike detection, even in the presence of strain [20,21]. The goal of this work is to quantitatively analyze the thermal response of embedded FBG sensors as a result of rapid temporal transients during rapid temperature changes and compare the results to previous models.

## 2. FBG High Temperature Theory

The center wavelength of the FBG reflected spectrum is called the Bragg wavelength $\lambda_{B}=2\Lambda n_{eff}$, where Λ is the FBG spatial period and $n_{eff}$ is the effective refractive index. Changes in temperature or strain cause shifts in the Bragg wavelength by altering Λ and $n_{eff}$. Neglecting the effects of strain, a shift in the Bragg wavelength $\Delta \lambda_{B}$ due to a change in temperature ΔT is given by

(1) $$\frac{\Delta \lambda_{B}}{\lambda_{B}}=\left(\frac{1}{\Lambda }\frac{d\Lambda }{dT}+\frac{1}{n_{eff}}\frac{dn_{eff}}{dT}\right)\Delta T.$$

During the FBG fabrication procedure (explained in the next section), electrons are excited by the UV fabrication laser and become trapped in a continuous distribution of energy states. When exposed to high temperatures, the FBG reflectivity decays as the trapped electrons are released. The detrapping rate due to thermal excitation depends on the FBG temperature as well as the depth of the trapped states, as described in greater detail in Reference [17].

**Figure 1:**
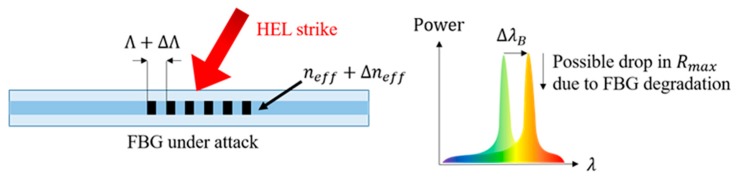
Spectral effects of HEL strike near an embedded FBG sensor.

Figure 1 illustrates the notional effects of a high-temperature HEL strike on the reflected spectrum of an FBG. The high temperatures created by the HEL strike cause a significant shift in $\lambda_{B}$ and can decrease the peak reflectivity $R_{max}$ of the FBG. FBG reflectivity decay that results at high temperatures during an HEL strike can be quantified using an integrated coupling constant (ICC) to characterize grating strength,

(2) $$ICC=\tanh^{-1}\left(\sqrt{1-T_{min}}\right),$$

where $T_{min}=1-R_{max}$ is the minimum transmission of the FBG (at $\lambda_{B}$) [17].

When normalized to its initial value [17], $ICC_{norm}$ can be approximately modeled by

(3) $$ICC_{norm}=\frac{1}{1+A_{0}exp(aT){\left(t/t_{1}\right)}^{\frac{T}{T_{0}}}}$$

where $A_{0}$, a, $t_{1}$, and $T_{0}$ are fit parameters; *t* is time; and T is the FBG temperature. Equation (3) was obtained by modeling the spectral decay of an FBG during annealing in a tube furnace [17]. For constant temperatures ranging from 100 °C to 550 °C, the spectral behavior of an FBG was accurately modeled using the values of $A_{0}$, a, $t_{1}$, and $T_{0}$ listed in Table 1.

The goal of these experiments is to determine if Equation (3) also applies when FBGs that are embedded in a composite experience non-uniform heating over much shorter timespans (less than 1 s), as would be experienced during a high energy laser strike.

## 3. FBG Fabrication

The fiber Bragg grating sensors that were tested and evaluated in these experiments were fabricated as follows [22]. Corning SMF-28 Ultra Optical Fibers were hydrogenated at 1000 psi for 1 week to photosensitize them to 244 nm light, after which they were stored in a ScienTemp Model 43-1.7A Refrigeration System at −20 °C to prevent loss of hydrogenation. The fibers were then cut into 60-cm segments and approximately 1 cm of coating was stripped from each segment’s midsection. Each photosensitive fiber core was then placed behind a 1059 nm phase mask and exposed to 140 mW light from a 244 nm Coherent Innova 300C MotoFreD Ion Laser. This created a ~1-cm long grating, with a spatial period of 529.5 nm, an effective refractive index of 1.448, and a nominal Bragg wavelength of 1533 nm. 

Each fiber was then annealed for 2 hours at 120 °C in a Fisher Scientific Isotemp Oven to remove any excess hydrogenation. The annealing process degraded the average peak reflectivity by 2.68 dB. A layer of acrylate protective coating was applied to the stripped midsection of each fiber using a Vytran PTR-200-XLR Fiber Re-coater. A total of 16 FBG samples were successfully fabricated using this method. Reflectivity data was collected for each FBG before and after the annealing process by connecting a wide-band light source to one end of the fiber and measuring the depth of the notch in the transmitted spectrum at the other end of the fiber. The minimum post-annealing transmission values of the 16 samples ranged from −5 dB to −34 dB, as measured at the Bragg wavelength of each grating. Three FBG samples with stable spectral measurements, reflectivity, and transmission values (listed in Table 2) were used for the HEL tests described in this research. 

## 4. Experimental Setup

**Figure 2:**
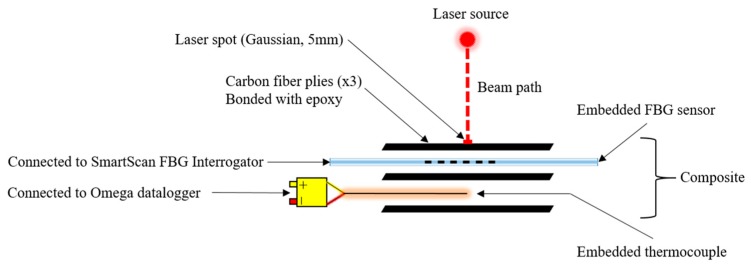
Arrangement of sensors embedded within a three-ply composite.

**Figure 3:**
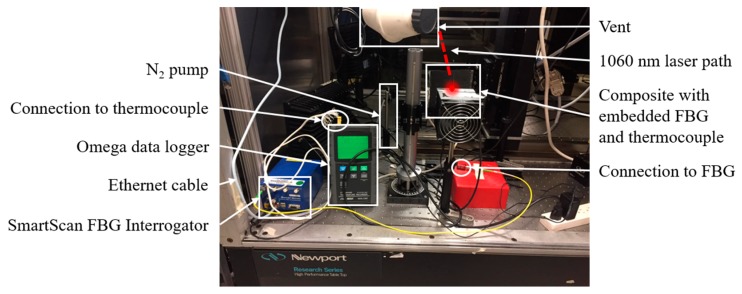
Experimental setup.

The objective of this research was to use a laboratory-scale HEL to emulate the effects of a directed energy weapon strike on a composite structure with embedded FBG sensors and to verify if previous models describing the decay of FBGs (in bare fiber) over long timespans still apply. Directed energy weapon strikes were emulated by irradiating the composite with an IPG Photonics YLM-100 1060 nm HEL (5 mm Gaussian beam diameter) at various power levels and for various amounts of time. To measure the temperature rise of the composite, a fiber Bragg grating and a Cr-Al thermocouple were embedded between different plies of a carbon fiber/epoxy composite as shown in Figure 2. The thermocouple was connected to an Omega RDXL12SD data logger and the fiber Bragg grating sensor was connected to a SmartScan Dynamic FBG Interrogator. The SmartScan is designed to return the peak wavelength of the reflected spectrum, but its User Data Protocol (UDP) was modified to measure and store full spectral data over time, which was then digitally sampled and organized into discrete wavelength bins (with resolution ~0.1 nm). A photograph of the experimental setup is shown in Figure 3.

Thermocouple temperatures and FBG spectral data were simultaneously collected during each of these HEL strikes. The temperature data logger monitored the thermocouple temperatures at 1 Hz, and the FBG was interrogated at a sample frequency of approximately 50 Hz, as defined by the rate in the SmartScan Interrogator. Because the coefficient of thermal expansion in the carbon fiber-epoxy composite is negligible [15], spectral shifts in $\lambda_{B}$ measured by the interrogator can be directly converted to temperature and expansion of the FBG due to thermally-induced strain in the composite can be ignored. During most of the HEL strikes, a controlled N_2_ flow was used to extinguish any flames that occurred. The FBG identifier, HEL power level, time on target, N_2_ usage, and level of FBG degradation for each test are listed in Table 3.

## 5. FBG Decay Results

**Figure 4:**
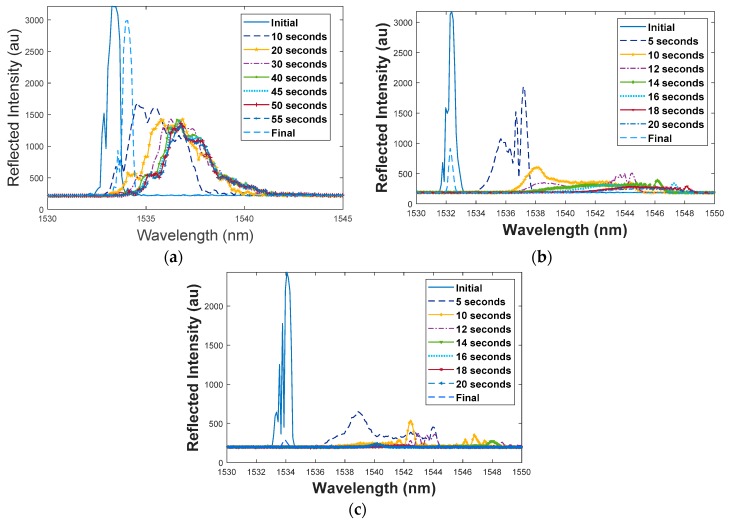
Two-dimensional plot of reflected spectrum over time during HEL tests: (**a**) FBG slightly degraded, (**b**) FBG moderately degraded, and (**c**) FBG severely degraded.

Various levels of degradation were observed due to FBG decay at high temperatures in the tests listed in Table 3. The reflected spectral data collected during these experiments is condensed into a series of two-dimensional plots of reflectivity vs. wavelength (shown in Figure 4). In each plot, there is a clear positive shift in $\lambda_{B}$ over time during the HEL strike, with a reflection peak that returns to the original Bragg wavelength after the strike ends. However, as the HEL strike heats the composite to high temperatures, the single, prominent peak in the reflected spectrum of the embedded FBG also splits, exhibiting several less significant sidelobes. This spectral splitting indicates that a non-uniform temperature profile is created by the 5 mm Gaussian HEL beam along the 10-mm length of the embedded FBG [16]. The degradation caused by the strike can be recognized by comparing the initial and final spectrum in the plots of Figure 4. In Figure 4b,c, when the HEL strike begins damaging the embedded FBG, the reflected spectrum decays to a highly unstable, barely detectable peak that fluctuates in wavelength. As can be seen in these plots, the reflected spectrum of the FBG is not well-defined and the Bragg wavelength is more difficult to precisely determine during HEL heating at high temperatures. Clearly, the reflectivity degradation is worse at higher HEL power levels and when N_2_ is not applied to extinguish the flames.

**Figure 5:**
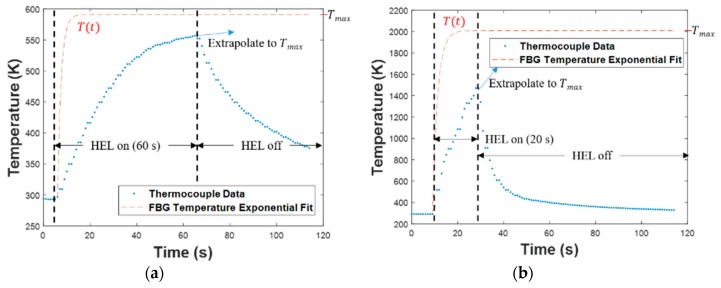

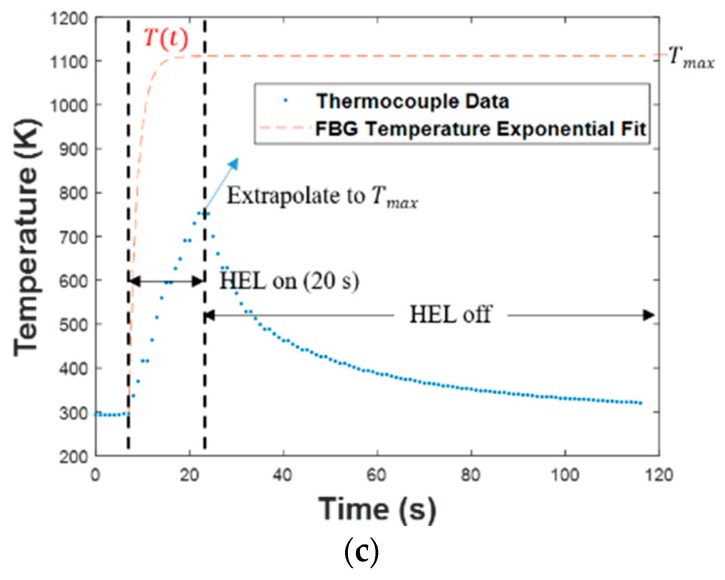
Thermocouple temperature data and exponential fit curves during HEL tests: (**a**) FBG slightly degraded, (**b**) FBG moderately degraded, and (**c**) FBG severely degraded.

To use Equation (3) in the context of these experiments, it is necessary to understand the temperature variation at the location of the FBG sensor. Figure 5 shows the temperature measured by the thermocouple as a function of time (blue curves). However, the FBG sensors, which were nearer to the front surface (as was shown in Figure 2), heated more rapidly than the thermocouples embedded near the back surface because of the proximity of embedded FBG sensors to the HEL strike [16,20]. Therefore, alternate exponential fit curves with smaller time constants $\tau_{T}$ were calculated (as described below) to model the FBG sensor temperature, as shown by the red dashed lines in Figure 5. The maximum temperatures of these fit curves were extrapolated to match the final temperature predicted by the exponentially increasing trend in the thermocouple temperature data. 

**Figure 6:**
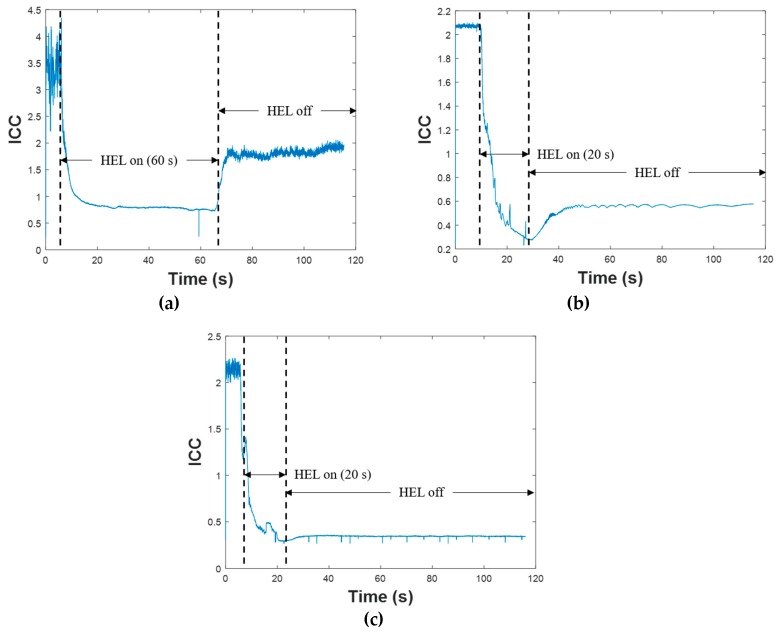
ICC data calculated using Equation (2) based on raw reflectivity data: (**a**) FBG slightly degraded, (**b**) FBG moderately degraded, and (**c**) FBG severely degraded.

**Figure 7:**
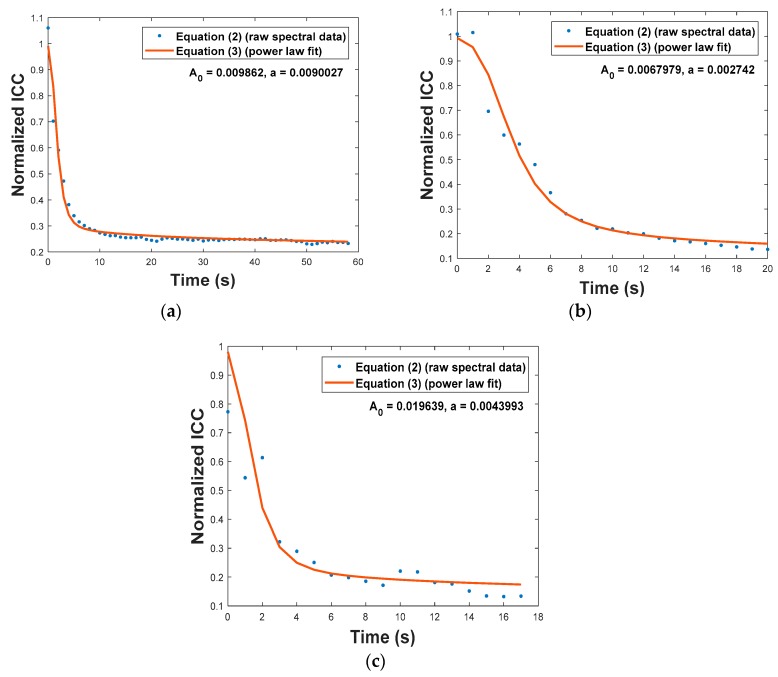
Normalized ICC decay of embedded FBG sensors during HEL strikes (recovery after tests not shown): (**a**) FBG slightly degraded, (**b**) FBG moderately degraded, and (**c**) FBG severely degraded.

The ICC of each FBG was then calculated using Equation (2) along with the peak reflectivity data collected by the FBG interrogator. These data are shown in Figure 6. The differences in initial ICC values are due to the differences in initial reflectivity (listed in Table 2). Since the value of the ICC is related to grating strength, the drop in ICC shown in Figure 6 during the laser strike indicates degradation in the reflectivity, whereas the increase in ICC after the HEL turns off demonstrates partial recovery in the FBG and increased reflectivity (most notable in Figure 6a since FBG A is only slightly degraded after the laser strike ends). The ICC data during the time period when the HEL is on (i.e., when heat is applied) were then normalized to the average value of the pre-strike ICC data. The normalized ICC data are plotted as blue dots in Figure 7.

In Reference [17], Equation (3) was used to accurately model the decay in reflectivity of bare FBGs during annealing over long timespans at a constant temperature. However, in the tests described here, the embedded FBG temperature increased quickly over much shorter timespans, and the temperature was not uniform over the length of the FBG. When the FBG temperature (labeled T(t) in each plot in Figure 5) is used in place of T in Equation (3), and the parameters $\tau_{T}$, $A_{0}$, and a are adjusted for best fit with the results based on Equation (2), the red curves plotted in Figure 7 and the fit parameters listed in Table 4 are obtained. Because Equation (3) does not model FBG recovery during cool-down, only the FBG heating curves are used for each fit and the time axes in Figure 7 are limited to the Time on Target values listed in Table 3. $T_{0}$ is set to 5250 K (the value obtained in Reference [17], used also in Reference [23]) and $t_{1}$ = 1 s (to match the time units in each plot). As can be seen in Figure 7, there is generally good agreement between the red curves based on Equation (3) using the fit parameters in Table 4 and the actual ICC data taken during an HEL strike, as indicated by the blue dots.

For these three representative tests, $\tau_{T}$ was within the interval 1.85 ± 0.25 s. These time constants are consistent with observations made in previous experiments [16,20]. The fit parameters $A_{0}$ and a obtained from Test 1 (slight FBG degradation) are the closest to the values obtained by Erdogan [17]. The other fit parameters are within one order of magnitude of Erdogan’s values. The accuracy of this fit might be improved with faster temperature data sampling. These results demonstrate that the power-law model in Reference [17] is reasonably applicable to high-temperature thermal decay of embedded FBG sensors caused by rapid, non-uniform heating, such as would occur during a HEL strike. 

## 6. Conclusions

The experiments described in this paper were conducted to characterize the rapid response of embedded FBG sensors to high temperatures that result during HEL strikes and to compare these results with previous research that models decay of bare FBGs during annealing. The degradation in the peak reflectivity and instability of the peak Bragg wavelength were consistent with results demonstrating a non-uniform temperature profile on an FBG sensor during an HEL strike [16]. Additionally, the power-law relationship proposed in Reference [17] for constant-temperature FBG decay during annealing in a tube furnace also applies to the decay of an embedded FBG sensor during rapid heating, such as would occur during a HEL strike. Further characterization of the reflectivity decrease during rapid FBG decay is the focus of ongoing research efforts. 

## Figures and Tables

**Table 1:** Previously obtained ICC fit parameter values [17].

Parameter	Value
$A_{0}$	(1.86 ± 0.22) × 10^−3^
a	(7.64 ± 0.19) × 10^−3^
$t_{1}$	1 min
$T_{0}$	5250 ± 250 K

**Table 2:** Initial Transmissivity of the Three FBG Samples Used in These Experiments.

FBG	Test	$T_{min}$ (dB)
A	1	−21.61
B	2	−11.93
C	3	−13.32

**Table 3:** FBG Testing Matrix.

FBG	Test	Laser Power (W)	Time on Target (s)	N_2_ Used?	FBG Degradation Level
A	1	25	60	Yes	Slight
B	2	100	20	Yes	Moderate
C	3	100	20	No	Severe

**Table 4:** ICC Fit Parameters for Tests 1–3.

	Test 1	Test 2	Test 3	Ref. [17]
Damage	Slight	Moderate	Severe	Varies
$\tau_{T}$	1.536 s	2.219 s	1.8232 s	N/A: constant T
$A_{0}$	9.86 × 10^−3^	6.80 × 10^−3^	1.96 × 10^−2^	(1.86 ± 0.22) × 10^−3^
a	9.00 × 10^−3^	2.74 × 10^−3^	4.40 × 10^−3^	(7.64 ± 0.19) × 10^−3^
